# Monitoring Visual Cortical Activities During Progressive Retinal Degeneration Using Functional Bioluminescence Imaging

**DOI:** 10.3389/fnins.2021.750684

**Published:** 2021-10-04

**Authors:** Darryl Narcisse, Sourajit M. Mustafi, Michael Carlson, Sanghoon Kim, Subrata Batabyal, Weldon Wright, Samarendra K. Mohanty

**Affiliations:** Nanoscope Technologies LLC, Bedford, TX, United States

**Keywords:** retinal degeneration, visually-evoked activity, functional imaging, bioluminescence, multi-characteristic opsin

## Abstract

Mouse models of inherited retinal degenerative diseases such as retinitis pigmentosa are characterized by degeneration of photoreceptors, which hinders the generation of signal to be transmitted to the visual cortex. By monitoring Ca^2+^-bioluminescence neural activity, we quantified changes in visual cortical activities in response to visual stimuli in RD10 mice during progression of retinal degeneration, which correlated with progressive deteriorations of electro-retinography signal from the eyes. The number of active neurons in the visual cortex, the intensity of Ca^2+^-bioluminescence response, and neural activation parameter showed progressive deterioration during aging. Further, we correlated the thinning of retina as measured by Optical Coherence Tomography with the decrease in visual cortical activities as retinal degeneration progressed. The present study establishes Ca^2+^-bioluminescence monitoring as a longitudinal imaging modality to characterize activities in visual cortex of retinal degenerative disease models and therapeutic interventions.

## Introduction

According to the World Health Organization, approximately 285 million people are estimated to be visually impaired worldwide (Pascolini and Mariotti, 2012). Retinal dysfunction due to photoreceptor degeneration as in Age-related macular degeneration or Retinitis Pigmentosa, leads to reduced signal transduction or transmission to the visual cortex (Burton, 2003). The current clinical trials for partial restoration of vision in blind patients with degenerated retinas involve retinal implants, transplants, or stem cells (Uy et al., 2013; Goetz and Palanker, 2016). In case of optic neuropathy or enucleation of the eye, stimulation of primary visual cortex by electrical and optogenetic methods is being attempted in preclinical and clinical trials to provide some level of vision. However, the effectiveness of these attempts depends on retinotopic mapping of visual cortex activities. Moreover, methods using implanted multielectrode arrays to record visually evoked activities in visual cortex are limited by poor resolution and scar-tissue formation around implanted electrodes (Leach et al., 2010; Prodanov and Delbeke, 2016). Therefore, there is a growing need to develop methods for non-invasive, spatio-temporal mapping of neural activities in the retina and visual cortex with high resolution to monitor and treat the progression of visual disorders.

The goal of this study is to deploy a non-invasive imaging tool for longitudinal monitoring of cortical activities during progressive degeneration of the retina. To address this goal, we used a newly developed Ca^2+^-sensitive bioluminescence assay instead of fluorescence to allow simultaneous long-term cortical imaging upon visual stimulation (Batabyal et al., 2018). In electing to use a methodology other than fluorescence, we simplified the recording hardware needed (having no need for excitation light and the associated filters) and avoided the risks of autofluorescence (confounding signal) and phototoxicity (destroying viable experimental neurons). We monitored simultaneous measurements of cortical activities during retinal photoreceptor degeneration in the RD10 mouse model. The Ca^2+^-bioluminescence response in the visual cortex was further correlated with electroretinography (ERG) signal from the eyes (Pinto et al., 2007; Benchorin et al., 2017). Further, loss of the photoreceptor layer and retinal thinning as monitored by Optical Coherence Tomography (OCT) (Huber et al., 2009; Giannakaki-Zimmermann et al., 2016) correlated well with the decrease in visually evoked cortical activities.

## Materials and Methods

### Viral Packaging of Fusion Constructs of MCO and Bioluminescent Probe

The MCOII gene was fused with GeNL-Ca^2+^ and synthesized using a DNA synthesizer and the sequence was verified. As calcium acts as a universal second messenger, the resulting construct would allow both indicative and operative functionality on a cellular level through the increased bioluminescence in response to calcium flux (indicative/reporter) reflecting cellular activity and optogenetic stimulation of cellular activity via opsin channel influx of calcium (operative/actuator) in response to light activation. Synthesized GeNL-Ca^2+^ fused MCOII plasmid (bMCOII) was cloned into pAAV MCS vector via its BamH1 and XhoI sites. We used the synthesized plasmid of bMCOII with CAG promoter and packaged it into Adeno-associated Virus AAV 2/5. AAV2/5 physical titers were obtained by quantitative PCR using primers designed to selectively bind the AAV inverted terminal repeats. The concentration of virus used was 1.23 × 1013 VG/ml.

### Mouse Preparation

Wild type (C57BL/6J) and retinal degenerated mice (B6. CXB1-Pde6brd10/J, Jackson Laboratories) of both sexes (2 Males and 3 Females) were used in the experiments reported here. The animals were obtained from Jackson laboratory and bred in the animal facilities of Nanoscope Technologies. The mice were maintained on a 12:12 (day: night) light cycle and were treated humanely in strict compliance with IACUC on the use of animals in research.

### Cranial Window Surgery

**FIGURE 1:**
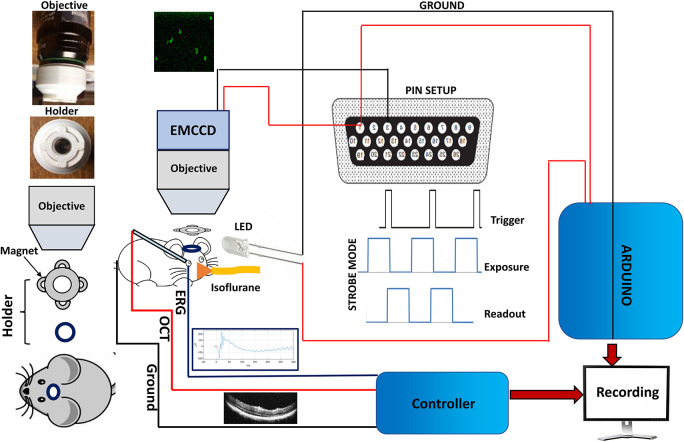
Setup for Ca^2+^-bioluminescence imaging and simultaneous measurements of ERG and SD-OCT in mice. NS-Neel: Experimental set up for simultaneous recording of Ca^2+^-bioluminescence signal in visual cortex and ERG/SD-OCT measurements in eye. The head mount setup ensures that recording in the same area in visual cortex is performed during longitudinal monitoring of visual evoked response.

At 3 weeks of age, mice were anesthetized with an intra-peritoneal injection of ketamine. After the animal was appropriately anesthetized, fur in the cranial area was removed chemically by administration of potassium thioglycolate. A midline incision through the cranial skin provided access to the skull. The attached nuchal musculature was retracted, and the periosteum was scraped away. A 4 mm^2^ window was opened over the right visual cortex using a dental drill. Four microliter of AAV2/5 virus (1.23 ×10^13^ GC/ml) was injected into 4-spots (1 μL each, separated by 2 mm) in the right visual cortex using a 33-gauge needle Hamilton syringe. The tip of the syringe was gently introduced into the cortical surface to avoid any bleeding. After 5 min of application of virus solution, 10 μl of antibiotic (ciprofloxacin) was applied over the cortical surface to prevent any infection. A PDMS-covered (∼100 μm thick) cortical mount (see Figure 1) was then placed over the surgical area and fixed with cyanoacrylate gel and secured with dental cement. Extreme care was taken so that the dental cement did not spread into the cortical window area. The area of the visual cortex was kept as clean and transparent as possible. Each surgery was done within 30 min after which the mouse was injected with 500 μl of saline injection via IP to dilute the effect of anesthesia. The mice were kept in the recovery cage for approximately for 2–3 h before being returned to cages separated from any other mice to prevent potential damage to the optical window.

### Ca^2+^-Imaging Assay in Anesthetized Mice

The mice were anesthetized by a slow flow of isoflurane (∼1%) and oxygen (4%), as starting proportion, via a mask inserted over the mouse’s snout. Before the start of the experiment, the cortical surgical area was washed with 100 μl of 1X PBS (pH 7.4) solution and 10 μl of 25 μM furimazine was directly added in the visual cortex. During the experiment, the mouse was monitored continuously for its vital signals. The proportion of the gas was adjusted to keep the animal anesthetized throughout the experimental session. The continuous images were recorded with an EMCCD camera with 25X/1.05 NA objective and 100 ms exposure time. The images were processed and quantified in ImageJ and further analyzed in Origin 2018. Various pulse width, intensity, and frequency of light stimulation settings were used to collect data. The camera exposure time was also varied from 50 to 200 ms and recording was done in deadtime mode. The detailed experimental setup has been shown in Figure 1. After surgical implantation of the optical mount, centered on the craniotomy window over the visual cortex, the transfected mouse was anesthetized using isoflurane and the protective PDMS covering was removed. Substrate was added and—using the optical mount as a guide—the camera was lowered into place with the interlocking optical mount and camera guide ensuring that the craniotomy window was centered and remained in place. Once in place, the mouse’s head could not be moved out of place without compromising the interlock between the optical mount and the camera guide. The substrate was allowed to permeate the tissues for 5 min before the experiment began. Tropicamide dilation solution was introduced to the eye which was to be the subject of stimulation and stimulating LEDs were placed close to the experimental eye.

### Artificial Intelligence Analysis on Neural Networks

Convolution neural network (CNN) (Ciresan et al., 2012) was used to evaluate neuronal communication in visually evoked cortical Ca^2+^-bioluminescence recording. The CNN algorithm consisted of four convolutions, four relu-activation function, four max-pooling and two fully connected layers. For training the CNN network, visually evoked GCaMP6 fluorescence images in mouse visual cortices were used. These training parameters were then applied to identify network architecture, including node and communication pathways in visually evoked Ca^2+^-bioluminescence recording. To quantify neuronal activities, Neuronal activation parameter (NAP) is defined as∑_*n*_*J*_*n*_(△*I*|*I*_0_)*_n_*, where “n” is number of nodes of signal communication between neurons, Jn is the number of connections associated with each node and ΔI/I_0_ is the fractional change in Ca^2+^-bioluminescence signal.

### Electroretinogram Recording Integrated With Spectral Domain Optical Coherence Tomography System

We performed Electroretinogram (ERG) recording and SD-OCT imaging using NS-Neel (Nanoscope Instruments Inc.). For visually evoked electrical measurement of retinal activities, mice were dark-adapted overnight (*N* = 5). For performing Electroretinogram experiments, mice were anesthetized using Isoflurane. Mice were placed on a heating pad set to ∼35°C. Their pupils were dilated using drops of tropicamide. The ground needle electrode was placed into the tail and a reference needle electrode was placed sub-dermally between the eyes. Silver wire contact lens electrodes were used as a recording electrode. The light stimulation intensity was varied from 0.01 to 10 cd-s/m^2^. For each stimulation intensity, 20 light-evoked electrical responses were recorded and averaged.

SD-OCT imaging of the eye was performed using a broadband super luminescent diode (λc = 840 nm, Δλ = 60 nm). SD-OCT is based on spectral domain implementation of the OCT system developed in the OCT research community and has been in clinical Ophthalmology practice. In the SD-OCT, the reference mirror is not scanned to perform depth scan. The interference signal between the reflected intensities from the reference mirror and the sample microstructures is detected with a spectrometer as a function of wavelength. The detected signal (as a function of wavelength) is then Fourier transformed to obtain an intensity profile as a function of depth. Thus, SD-OCT scans the whole depth of the sample without any mechanical scanning. Prior to imaging, mice were anesthetized. Mice were placed on the platform of the SD-OCT and retinal thickness measurements were made.

### Statistical Analysis

Data are expressed as the Average (Av.) ± Standard deviation (SD). The data at different time points were analyzed by a two-tailed Student’s *t-*test. A *p*-value of < 0.05 was deemed significant.

## Results

To understand progressive retinal degeneration, we monitored the Ca^2+^-bioluminescence response in the visual cortex of RD10 mice model upon visual stimulation with varying stimulation parameters (light intensities: 13–22 μW/mm^2^; pulse width 10–30 ms). Figure 1 shows the experimental set up for simultaneous recording of Ca^2+^-bioluminescence signal in visual cortex and ERG/SD-OCT measurements in the eye. To achieve longitudinal imaging of Ca^2+^-bioluminescence activities in the same area of the visual cortex over repeated measurements, a magnetically coupled head stage and camera guide. The optical mount on the mouse and the camera guide on the EMCCD form the two interlocking halves of the holder assembly which ensure the optical window remains centered during the experiment. The permanent surgical implantation of the optical mount centered around the craniotomy window allows repeated longitudinal recordings fixed on the same area. Time series images of visually evoked Ca^2+^-bioluminescence response in visual cortex was recorded using an EMCCD camera. Along with the Ca^2+^-bioluminescence signal, we also simultaneously monitored ERG response and SD-OCT images of retina using the setup described in Figure 1.

### RD10 Mice Exhibited Primary and Secondary Visual Cortex Ca^2+^-Bioluminescence Responses of Progressively Weaker Intensity With Degeneration of Retina

**FIGURE 2:**
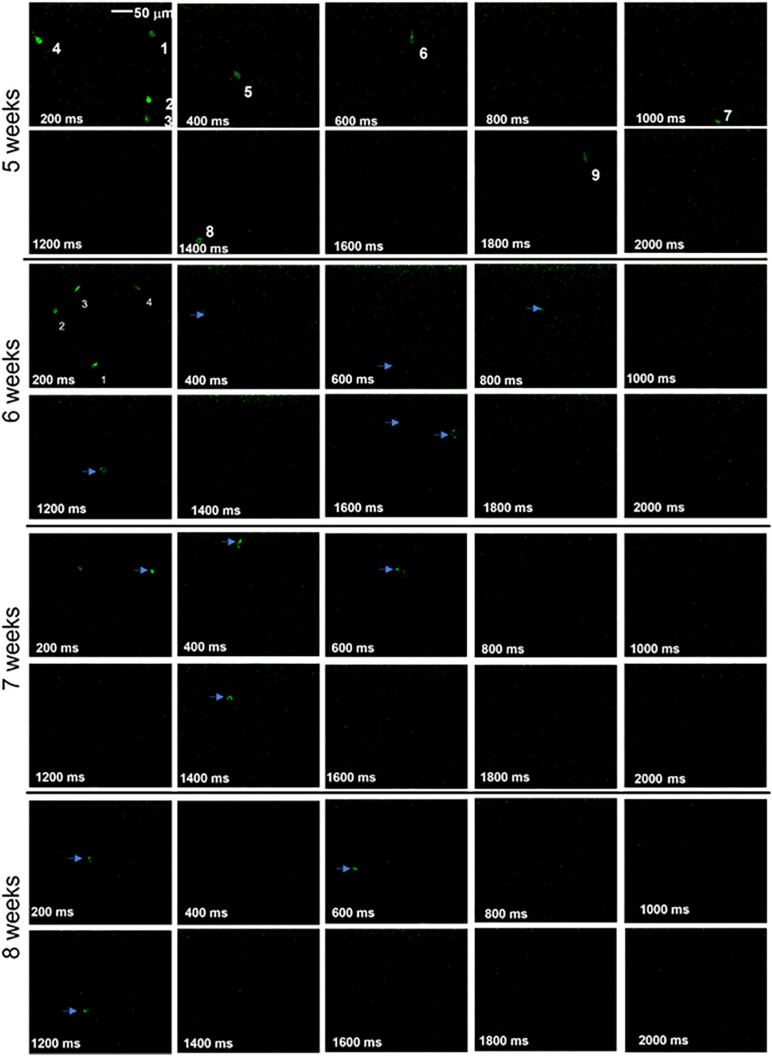
Ca^2+^-bioluminescence profile for RD10 mice upon visual stimulation. Time-lapse visually evoked Ca^2+^-bioluminescence images of RD10 mice primary visual cortex at 5, 6, 7, and 8 weeks. Arrows and numbers indicate specific neurons activated during visual stimulation which were tracked during the experimental session. Scale: 50 μm.

The time lapse images of Ca^2+^-bioluminescence response in visual cortex of RD10 mice is shown in Figure 2. The Ca^2+^-bioluminescence response in the visual cortices of RD10 mice is shown for 5–8 weeks of age with a 1-week measurement interval. During 5th week of life, upon visual stimulation some neuronal activities are immediately observed in the primary visual cortex (V1). These responses, appearing within 500 ms of visual stimulation are henceforth referred to as primary response. Ca^2+^-bioluminescence responses were also observed from 1,000 to 2,000 ms after light stimulation and are referred to as secondary response. At 5 weeks of age, in response to visual stimulation several neurons showed activities between 0 and 500 ms (primary) while some activities were observed at > 1 s (secondary). In successive recordings (6, 7, and 8 weeks) there was a clear, successive decrease in V1 activities as measured by the Ca^2+^-bioluminescence intensity of the primary response. We measured the baseline Ca^2+^-bioluminescence response (without light stimulation) in both wild type and RD10 mice and compared them with the visually evoked response. Representative time-lapse images of Ca^2+^-bioluminescence response in primary visual cortex of wild type mice for 6 and 7 weeks of age is shown in Supplementary Figure 1. Unlike RD10 mice, the wild type mice show consistent visually evoked neuronal activities in V1 with age.

### Number of Active Neurons, Fractional Intensity, Fidelity, and Neuronal Activation Parameter All Show Progressive Degradation in RD10 Mice Over Time

**FIGURE 3:**
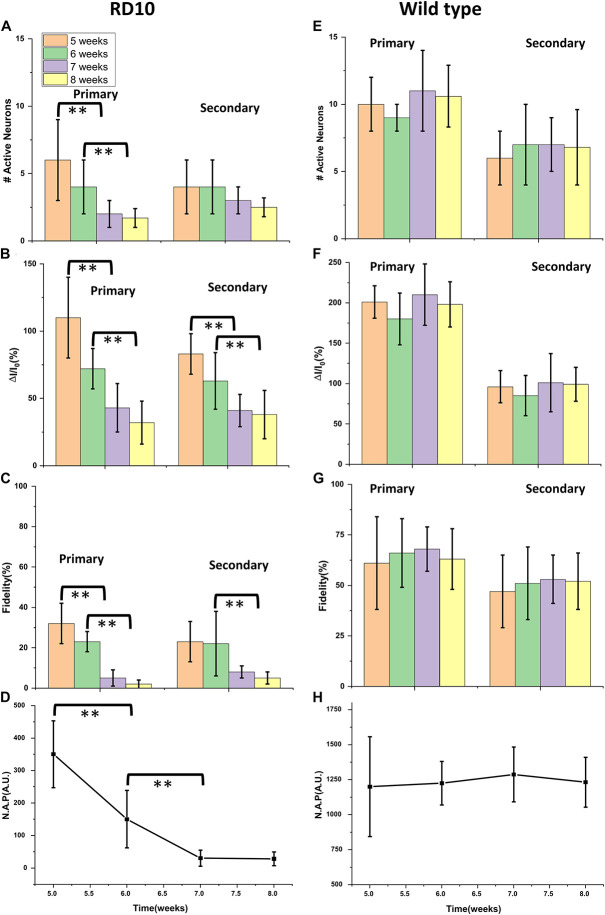
Longitudinal decay of Ca^2+^-bioluminescence activities in bMCOII sensitized visual cortex of RD10 mice with age. Decrease of Ca^2+^-bioluminescence response of RD10 mice. The longitudinal decay of number of active neurons **(A)**, the fractional change in Ca^2+^-bioluminescence intensity **(B)**, the fidelity **(C)** and Neuronal Activation Parameter (N.A.P.) **(D)** for primary and secondary neurons are shown. Similar profile of active neuron number **(E)**, the fractional change in Ca^2+^-bioluminescence intensity **(F)**, Fidelity **(G)** and Neuronal Activation Parameter (N.A.P.) **(H)** in wild type mice is shown. *N* = 5, Av. ± SD, Student *t*-test was performed with **p < 0.01.

Progressive decay of Ca^2+^-bioluminescence activity, especially in the primary response, clearly correlates with age. We defined active neurons as neurons in layer 2 that showed detectable increase in Ca^2+^-bioluminescence in response to visual stimulation, which was absent during no-stimulation period (baseline). The longitudinal decay in the number of active neurons (Figure 3A), fractional change in Ca^2+^-bioluminescence intensity (Figure 3B), the fidelity (Figure 3C) and Neuronal Activation Parameter (N.A.P.) (Figure 3D) showed progressive decrease with aging in RD10 animals. Similar comparison in wild type animal for number of active neurons (Figure 3E), the fractional change in Ca^2+^-bioluminescence intensity (Figure 3F), fidelity (Figure 3G) and Neuronal Activation Parameter (N.A.P.) (Figure 3H) shows very little variation during the age range of 5–8 weeks.

The histogram plot in Figure 3A represents the subsequent decrease in the number of primary active neurons involved in visually evoked response from 5 to 8 weeks of age. The number of secondary neurons seems to decrease with age progression, though analysis does not show this trend to be statistically significant. The fractional Ca^2+^-bioluminescence intensity shows significant decay both for primary and secondary response during progression of retinal degeneration in RD10 mice (Figure 3B). The fidelity of both primary and secondary response likewise shows a progressive decrease from 5 to 8 weeks of age (Figure 3C). The artificial intelligence assisted Neural Activation Parameter (N.A.P.), which measures the strength of signaling networks, shows subsequent decrease in value and reaches baseline at the age of 7 weeks (Figure 3D). As control data we measured the number of active neurons (Figure 3E), fractional increase in Ca^2+^-bioluminescence signal (Figure 3F), fidelity (Figure 3G) and neural activation parameter (N.A.P.) (Figure 3H) in wild type mice from 5 to 8 weeks of age. The above parameters remain unchanged during the above time frame.

### Ca^2+^-Bioluminescence Signal of RD10 Mice at 7–8 Weeks Is Comparable to Baseline While Remaining Robust in Wild Type

**FIGURE 4:**
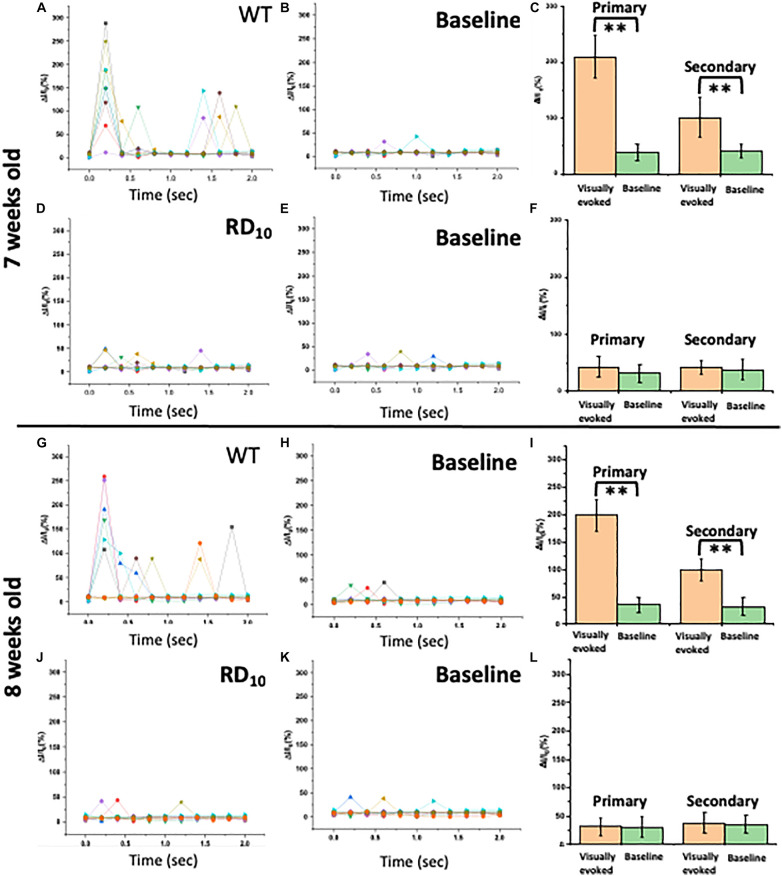
Characterization of Ca^2+^-bioluminescence signal of Wild type and RD10 mice. Fractional increase in Ca^2+^-bioluminescence intensity upon light stimulation **(A)** and without stimulation **(B)** in 7 weeks old wild type mice are shown. **(C)** The corresponding comparison of average Ca^2+^-bioluminescence intensity with the baseline values. The fractional increase in Ca^2+^-bioluminescence intensity upon light stimulation **(D)** and without stimulation **(E)** in 7 weeks old RD10 mice. **(F)** The corresponding comparison of Ca^2+^-bioluminescence intensity with the baseline values. Fractional increase in Ca^2+^-bioluminescence intensity upon light stimulation **(G)** and without stimulation **(H)** in 8 weeks old wild type mice are shown. **(I)** The corresponding comparison of Ca^2+^-bioluminescence intensity with the baseline values. The fractional increase in Ca^2+^-bioluminescence intensity upon light stimulation **(J)** and without stimulation **(K)** in 8 weeks old RD10 mice. **(L)** The corresponding comparison of Ca^2+^-bioluminescence intensity with the baseline values. *N* = 5, Av. ± SD, ^∗∗^*p* < 0.01.

For 7–8 weeks old wild type mice, the visually evoked response in visual cortex upon light stimulation is threefold higher than the baseline level (without any light stimulation). Fractional increase in Ca^2+^-bioluminescence intensity upon light stimulation (Figure 4A) and without stimulation (Figure 4B) in 7 weeks old wild type mice are shown. The corresponding comparison of visually evoked Ca^2+^-bioluminescence intensity with the baseline values reveal significantly high visually evoked response in wild type mice upon light stimulation (Figure 4C). Correspondingly, in 7 weeks old RD10 mice the visually evoked response is comparable with its baseline activities. Fractional increase in Ca^2+^-bioluminescence intensity upon light stimulation (Figure 4D) and without stimulation (Figure 4E) are shown in RD10 mice. Comparing the average Ca^2+^-bioluminescence intensity in RD10 mice upon light stimulation and without stimulation shows no significant difference (Figure 4F). Fractional increase in Ca^2+^-bioluminescence intensity upon light stimulation (Figure 4G) and without stimulation (Figure 4H) in 8 weeks old wild type mice are shown. The corresponding comparison of average Ca^2+^-bioluminescence intensity with the baseline values (Figure 4I) shows threefold increase in Ca^2+^ -bioluminescence signal upon light stimulation. For corresponding 8 weeks old RD10 mice analysis of fractional increase in Ca^2+^-bioluminescence intensity upon light stimulation (Figure 4J) and without stimulation (Figure 4K) are plotted. The comparison of average Ca^2+^-bioluminescence intensity with the baseline values (Figure 4L) shows no significant increase in Ca^2+^-bioluminescence signal upon light stimulation with respect to baseline response. These results suggest complete loss of visual sensation in RD10 mice.

### Longitudinal Decay Profile of Ca^2+^-Bioluminescence Signal in RD10 Mice

**FIGURE 5:**
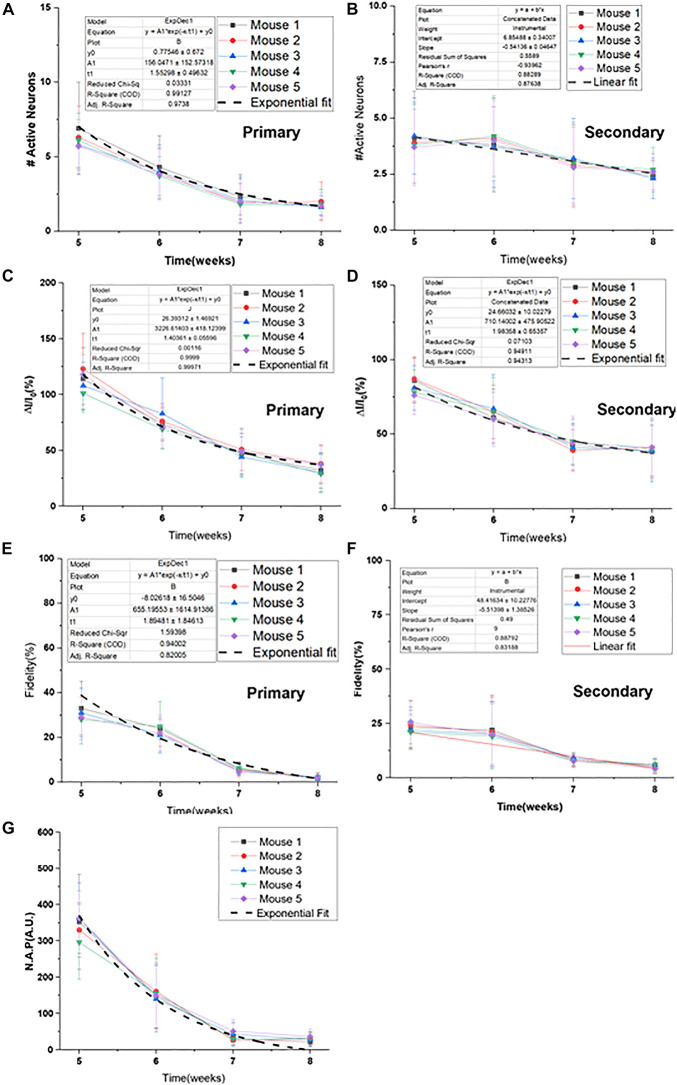
Longitudinal decay profile of Ca^2+^-bioluminescence signal in RD10 mice. Decay profile and corresponding exponential fit of primary **(A)** and secondary active neurons **(B)**. Decrease in fractional Ca^2+^-bioluminescence intensity and the fitted profile for primary **(C)** and secondary **(D)** response are shown. Fidelity measure and corresponding decay fit in primary **(E)** and secondary **(F)** response are depicted. Decrease in Neuronal Activation Parameter (N.A.P.) and corresponding fitted profile are shown in **(G)**. The associated exponential decay equations are listed with a “1” next to the plots. *N* = 5, Av. ± SD.

The longitudinal decay profile of each RD10 mouse is shown in Figure 5. The synergic decay of number of primary active neurons fit consistently with exponential decay profile (Figure 5A). However, the decay profile for secondary neurons shows a linear pattern (Figure 5B). The fractional decay of Ca^2+^-bioluminescence intensity of both primary and secondary responses reveals an exponential decay profile (Figures 5C,D). The fidelity in primary response and corresponding exponential decay fit are shown in Figure 5E. However, the fidelity decrease in the corresponding secondary response is also linear in nature (Figure 5F). The decay in neuronal activation parameter (N.A.P.) shows exponential nature with time (Figure 5G). The number of secondary neurons does not decrease significantly from 5 to 6 weeks hence the linear pattern of the profile for decrease in number of secondary responses. Table 1 details the fitted values for all the experimental parameters. For more accurate control we also monitored the baseline activities of RD10 mice without light activation. Interestingly, the baseline activities remain unchanged for RD10 mice as they age. Supplementary Figure 2 shows similar baseline activities in RD10 mice at 5 and 7 weeks of age.

**TABLE 1:** The Ca^2+^-bioluminescence activity profile in RD10 mice.

Parameter	Time constant (week)	Error (week)	Fit	Adj. R^2^
Active neuron (primary)	1.55	0.5	Exponential	0.9738
Fractional intensity (primary)	1.4	0.06	Exponential	0.99971
Fractional intensity (secondary)	1.98	0.65	Exponential	0.94313
Fidelity (primary)	1.89	1.85	Exponential	0.82005
NAP (A.U.)	1.16	0.01	Exponential	0.9985
Active neuron (secondary)	0.54 (slope)	0.046	Linear	0.8764
Fidelity (secondary)	5.51 (slope)	1.28	Linear	0.8313

*The fitted decay parameters of Ca^2+^-bioluminescence signal in RD_10_ mice.*

### Frequency and Intensity Dependence of Ca^2+^-Bioluminescence Signal in RD10 Mice

Amplitude and width of stimulus is varied from 10 to 22 μW/mm^2^ and pulse width is varied from 10 to 30 ms. The experimental parameters like number of active primary and secondary neurons, fractional increase of Ca^2+^-bioluminescence intensity, fidelity in primary and secondary response and neural activation parameter. The variation of these parameters on RD10 mice is shown in Supplementary Figure 3 and for wild type mice in Supplementary Figure 4. For 5 weeks old RD10 mice, the increase in intensity and pulse width leads to an increase in the number of active primary neurons (Supplementary Figure 3A) and fractional increase in Ca^2+^-bioluminescence signal (Supplementary Figure 3B). The fidelity and neuronal activation parameter also show similar increases (Supplementary Figures 3E–G). In the wild type model, no such sharp increasing trend is observed (Supplementary Figure 4).

### Visual Cortex Activity Changes in Ca^2+^-Bioluminescence Correlate With Electroretinography and Optical Coherence Tomography Data in RD10 Mice

**FIGURE 6:**
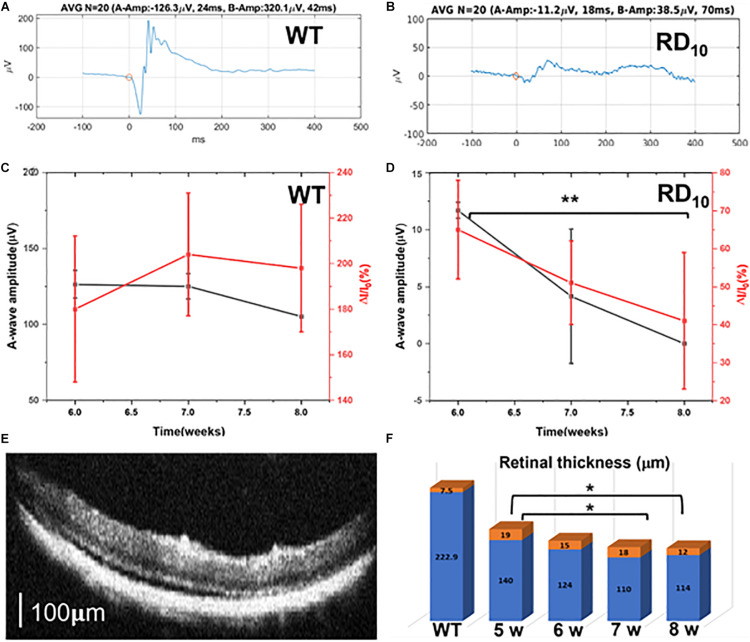
Correlation of Ca^2+^-bioluminescence signal with ERG and OCT data in RD10 mice. ERG signal of wild type **(A)** and RD10 **(B)** mice at 6 weeks of age. Correlation of the amplitude of A-wave and fractional Ca^2+^-bioluminescence intensity for Wild type **(C)** and RD10 mice **(D)**. **(E)** Representative OCT picture of RD10 mice retina at 7 weeks of age. Scale: 100 μm. **(F)** The progressive decrease of retinal thickness in RD10 mice with aging, from 5 to 8 weeks, mean (blue) + standard deviation (orange). *N* = 5, Av. ± SD, *p < 0.05, **p < 0.01.

As expected, upon comparison of the ERG signals of wild type (Figure 6A) and RD10 (Figure 6B) mice at 6 weeks of age, the ERG response for wild type animals is much higher than the response in RD10 mice. Correlation of amplitude of A-wave and fractional Ca^2+^-bioluminescence intensity for wild type (Figure 6C) and RD10 mice (Figure 6D) are shown. The RD10 animals show a decrease in Ca^2+^-bioluminescence intensity correlating with the decrease in A-wave amplitude. The representative OCT picture of RD10 mice retina at 7 weeks of age is shown in Figure 6E. The progressive decrease in retinal thickness in RD10 mice relative to wild type (Supplementary Figure 5A) between 5 and 8 weeks of age are depicted in Figure 6F and Supplementary Figures 5B–E.

## Discussion

Mouse models of retinitis pigmentosa (RP) are essential tools in the pursuit of fully understanding what cell types and processes underlie the degeneration observed in RP. Knowledge of these processes is required if we are to develop successful therapies to treat this currently incurable disease (Phillips et al., 2010). In the first stage of the disease, RD10 mice exhibited significant photoreceptor degeneration evident as a large reduction in outer nuclear layer and outer retinal thickness. As shown by earlier SD-OCT studies, the EZ(IS/OS) zone is much narrower and less intense in degenerated retinas when compared with WT controls (Oh et al., 2014; Garcia-Delgado et al., 2018). The EZ (IS/OS) zone was completely absent in Stage II RD10 mice indicating further progression of photoreceptor degeneration (Lu et al., 2013). As photoreceptors degenerate, light-dependent OCT changes in outer retina were also significantly diminished (Li et al., 2018). However, retinal pigment epithelium (RPE) cells themselves are not intrinsically sensitive to light, and only photoreceptors can initiate light responses in the outer retina (Fuhrmann et al., 2014; Volland et al., 2015; Shen et al., 2017; Tanabu et al., 2019). Activated photoreceptors are therefore likely to release signals that influence fluid transport in RPE cells in a light-dependent manner. Reduced light-dependent OCT changes in the outer retina in stage I RD10 mice are consistent with diminished photoreceptor signal in these animals (Li et al., 2018). As retinal degeneration progresses in RD10 mice, subretinal fluid typically accumulates and becomes pathological, first in smaller regions in the posterior pole (stage II) and then in extensive contiguous areas later in degeneration (stage III) (Sparrow et al., 2010). Earlier studies of electrophysiology of retinal ganglion cells (RGC) in RD10 mice show that there is a strong phase-locking tendency between the spectral peak of bursting RGC spikes (∼5 Hz) and the first peak of oscillatory field potential (∼5 Hz) across different age groups (Ferrari et al., 2011).

In the present study, rather than monitoring the activities in the retina, we characterized visually evoked neural activities in the visual cortex. Monitoring activities in the visual cortex is important for ultimate development of cortical prosthetics. In patients with complete optic nerve damage, vision restoration can only be achieved by cortical prosthetics (SGoo et al., 2011). The significant advantages of the cortical approach over its counterparts are the reduced power requirements, more predictable phosphene generation, less phosphene interaction, absence of flicker, and packing of more stimulation channels to increase resolution (Schwartz, 2004; Mirochnik and Pezaris, 2019). Our present study correlates losses in the photoreceptor layer of the retina and diminishing of the visually evoked response in the visual cortex. We also monitored any residual activities in the visual cortex even when there is complete loss of photoreceptors. We found no significant residual activity. At 7–8 weeks, when the photoreceptor layers are completely degraded as monitored by SD-OCT images, we observe that there is no significant difference between visually evoked neural activities as compared to baseline response without light stimulation.

The Ca^2+^-bioluminescence multi-characteristic opsin bMCOII (Ryu and Shenoy, 2009) is an ideal probe to quantify loss of visual activities in higher visual areas. The visually evoked Ca^2+^-bioluminescence signal due to neural activities also correlated well with electroretinogram (ERG) signal in the retina. For wild type mice, the intensity of Ca^2+^-bioluminescence signal in the visual cortex remains constant as does the amplitude of ERG a-wave. In RD10 mice there is concomitant decrease of a-wave amplitude and corresponding Ca^2+^-bioluminescence signal. As the photoreceptor layers decrease in thickness, the neural network structure in the visual cortex as measured by Artificial Intelligence assistant neural activation parameter (N.A.P.) shows a decrease until 7 weeks when no network connection is observed. In wild type animals, the N.A.P. is significantly higher—indicating the normal range of network connection—which highlights the already notable decrease in connection in RD10 mice even in early development. The animals were transfected at 3 weeks of age and it takes approximately 2 weeks for the AAV2/5 virus to express bMCOII (Ryu and Shenoy, 2009), hence we could only measure the visually evoked response from 5 weeks onwards. During the recording time (5–8 weeks of age) the decay constant of degradation of cortical response is 1.2–2. Interestingly, at 5 weeks of age in RD10 mice increasing the strength and duration of visual stimuli enhances the activities in the visual cortex. This phenomenon was absent for wild type mice. The intensity range of 10–20 μW/mm^2^ may be close to saturation limit for wild type animals, thus variation of intensity in this range does not evoke a large variation in cortical response. However, in RD10 mice due to degradation of the photoreceptive layer this may not be the case.

## Conclusion

The RD10 mouse is a unique model to monitor late onset retinal degeneration. In the present study we quantified changes in visual cortical activities in RD10 mice during progression of retinal degeneration by monitoring Ca^2+^-bioluminescence neural activity images in response to visual stimuli and compared this with the expected and typical progressive deteriorations of electroretinography (ERG) signal from the eyes. A strong correlation was found between the decrease of photoreceptive layer thickness in the retina and loss of neural activities in the visual cortex. The bMCOII sensitized visual cortex shows promise as a unique assay to measure changes in visual cortical activity. This experiment supports the value of bioluminescence as method for tracking long term cellular activity by avoiding the autofluorescence, phototoxicity, and lower resolution from fluorescence and electrical methods currently available. This should allow active long-term analysis of visual cortex activity changes secondary to optical disease such as retinal degeneration. There have been several optogenetic molecules which can sensitize the Retinal Ganglion Cell (RGC) layer to restore partial vision to subjects suffering from complete loss of photoreceptor layers. Such optogenetic molecules have shown promise in vision restoration (Freeman et al., 2011; Tochitsky et al., 2017). It will be interesting to see recovery of cortical neural activities in blind animal by sensitizing the retina by therapeutic optogenetic molecules (Wright et al., 2017) and monitoring visually evoked responses in visual cortex transduced by bMCOII.

## Data Availability Statement

The original contributions presented in the study are included in the article/Supplementary Material, further inquiries can be directed to the corresponding author/s.

## Ethics Statement

All experimental procedures were conducted according to the Nanoscope Technologies’ Institutional Animal Care and Use Committee approved protocol. This study involving animals was reviewed and approved by the Nanoscope IACUC.

## Author Contributions

DN, SMM, MC, and SKM carried out stimulation, imaging, and electrical recording experiments on cortical slices. SB, MC, and SMM carried out ERG experiments. DN carried out cortical injections and optical window implant. DN, SMM, and MC conducted *in vivo* bioluminescence activity imaging. SK performed OCT imaging. SMM performed AI analysis. SM supervised the project. All authors participated in planning of experiments, discussion, and data analysis and contributed to the preparation of the manuscript.

## Conflict of Interest

DN, SMM, MC, SK, SB, WW, and SKM were employed by the company Nanoscope Technologies LLC. SKM has equity interest in Nanoscope Technologies, LLC, which is developing products in biomedical diagnostics and therapeutic technologies.

## Publisher’s Note

All claims expressed in this article are solely those of the authors and do not necessarily represent those of their affiliated organizations, or those of the publisher, the editors and the reviewers. Any product that may be evaluated in this article, or claim that may be made by its manufacturer, is not guaranteed or endorsed by the publisher.
