# Supplementary material for: Monitoring Visual Cortical Activities During Progressive Retinal Degeneration Using Functional Bioluminescence Imaging
Source: Front Neurosci. 2021 Oct 4;15:750684. doi: 10.3389/fnins.2021.750684 (PMC8530108; doi:10.3389/fnins.2021.750684)
Supplement: Supplementary file 1 [file Data_Sheet_1.DOCX]

Supplementary Material

**Monitoring Visual Cortical Activities during Progressive Retinal Degeneration using Functional Bioluminescence imaging**

Darryl Narcisse^1†^, Sourajit Mustafi^1†^, Michael Carlson^1^, Sang Hoon Kim^1^, Subrata Batabyal^1^, Weldon Wright^1^, Samarendra Mohanty^1†^*

*Nanoscope Technologies LLC, Bedford, TX 76022, USA.*

* Correspondence should be addressed to S.M (smohanty@nanoscopetech.com)


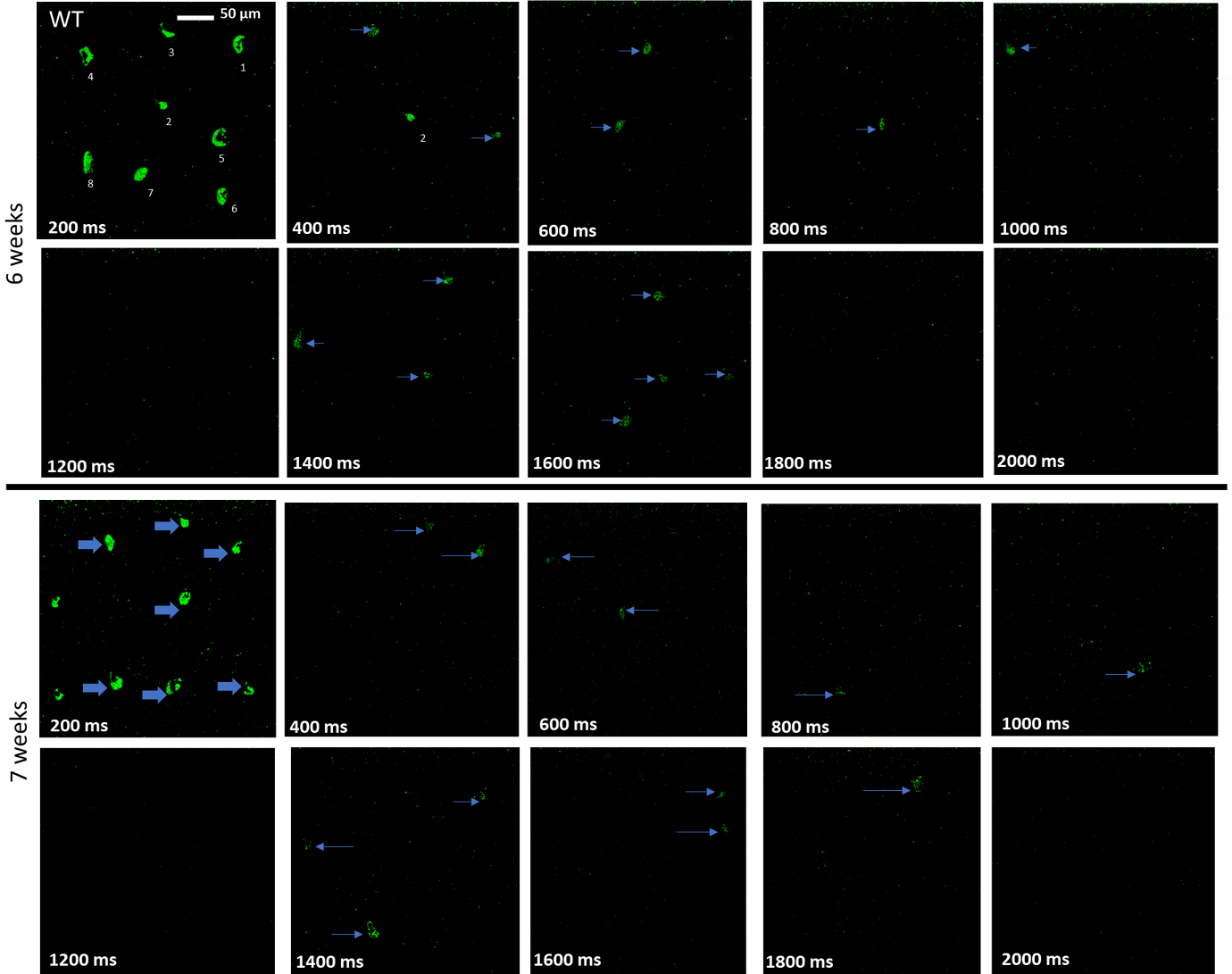


Fig S1. Visually-evoked Ca^2+^-bioluminescence response in primary visual cortex of Wild type mice. Top 2 rows: 6 weeks old; bottom two rows: 7 weeks old. The time-series images show response in V1 after visual stimulation of one eye. Arrows and numbers indicate specific neurons activated during visual stimulation which were tracked during the experimental session.


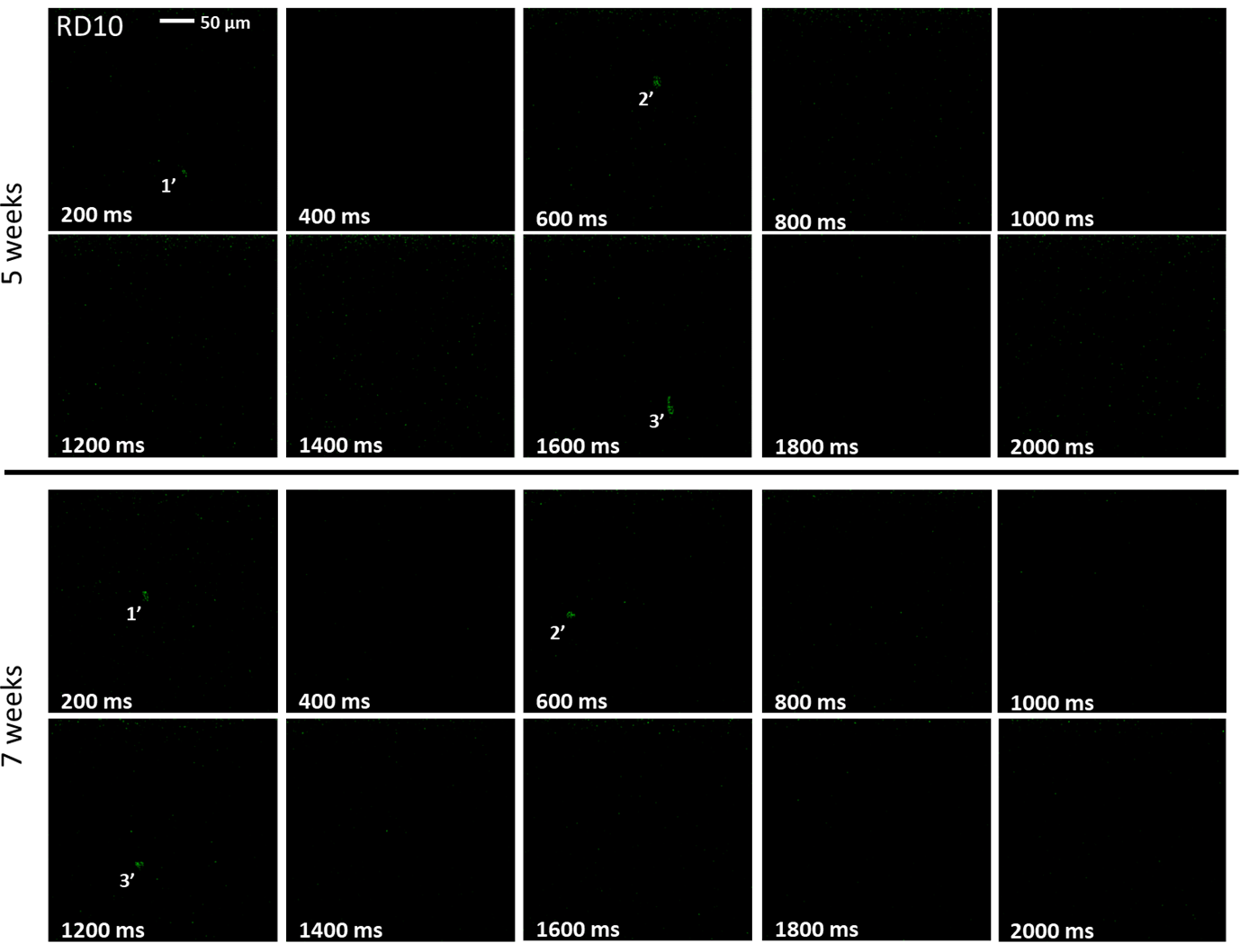


Fig S2. Representative baseline (with no light stimulation) Ca^2+^ -bioluminescence response in visual cortex of RD10 mouse. Top two rows: 5 weeks old; and Bottom two rows: 7 weeks old mouse. The time-series images show response in visual cortex after visual stimulation of one eye.

**
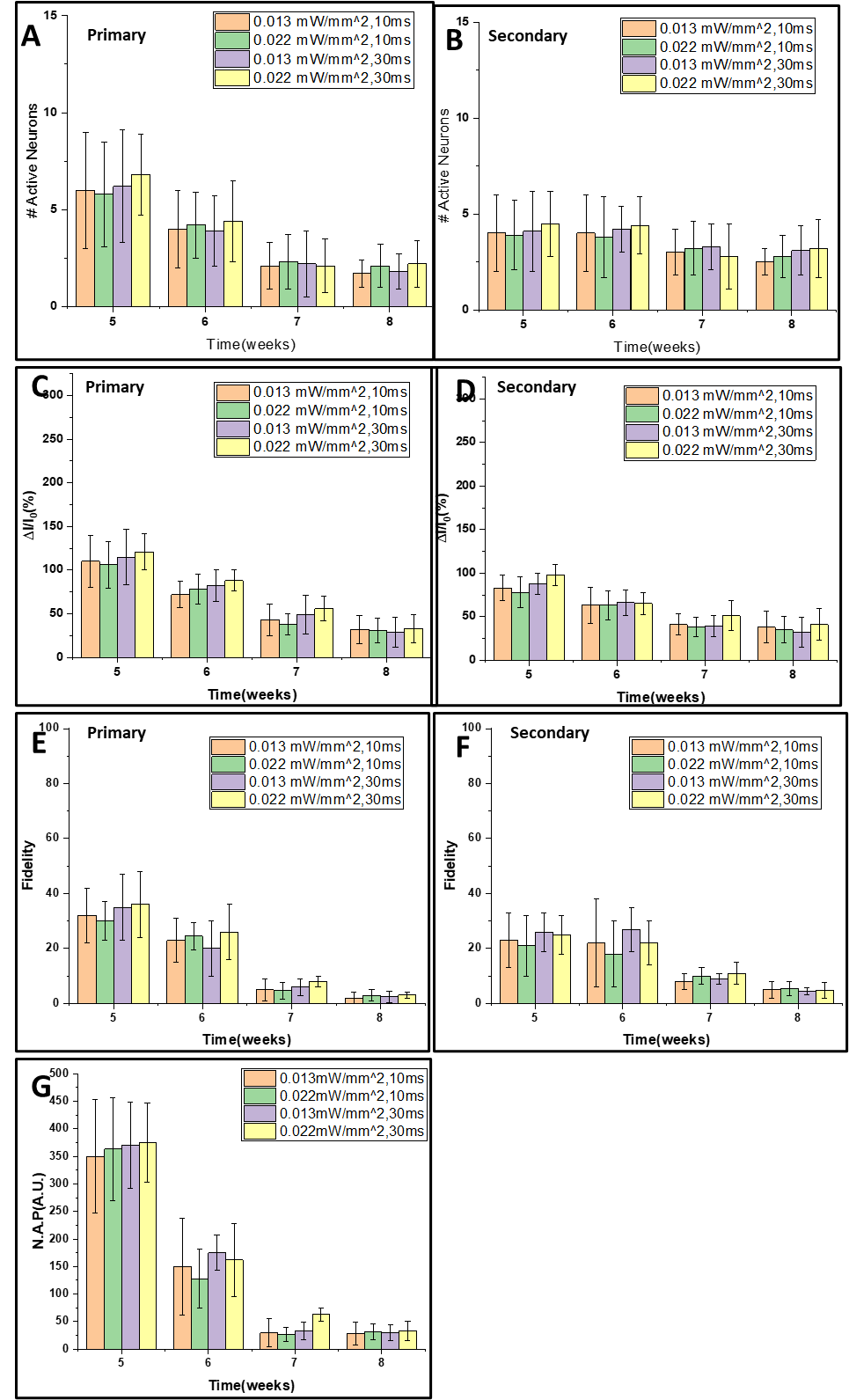
**

Fig S3. Dependence of Ca^2+^-bioluminescence signal in RD10 mice with respect to variation in light intensity and pulse width. (A) The number of primary neurons, (B) the number of secondary active neurons, fractional increase of Ca^2+^-bioluminescence intensity in primary (C) and secondary (D) response, the fidelity in primary (E) and secondary response (F). The decrease in Neural Activation Parameter (N.A.P.) is shown in G.


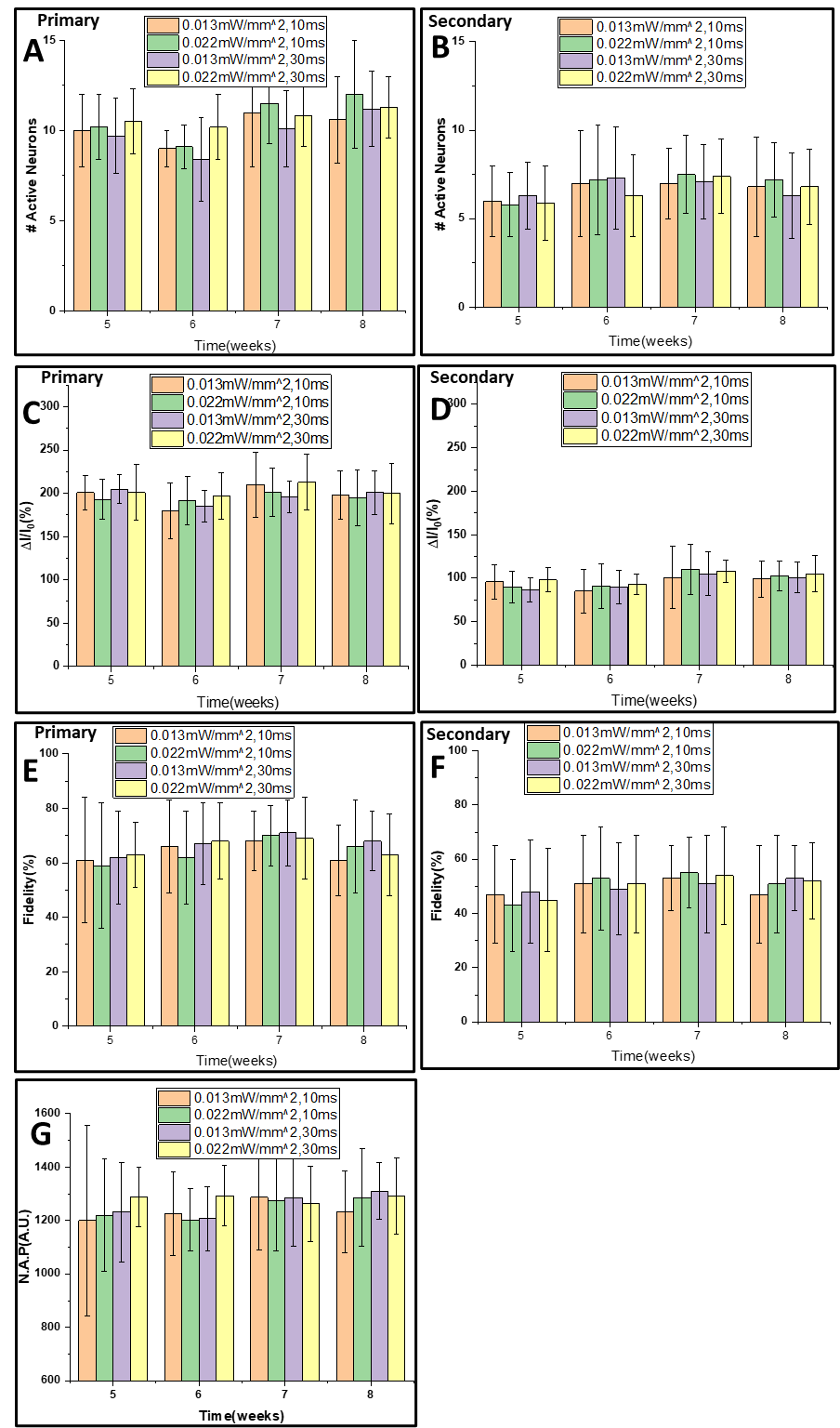


Fig S4. Dependence of Ca2+-bioluminescence signal in wild type mice with respect to variation in light intensity and pulse width. (A) The number of primary neurons, (B) the number of secondary active neurons, fractional increase of Ca2+-bioluminescence intensity in primary (C) and secondary (D) response, the fidelity in primary (E) and secondary response (F). The decrease in Neural Activation Parameter (N.A.P.) is shown in G.


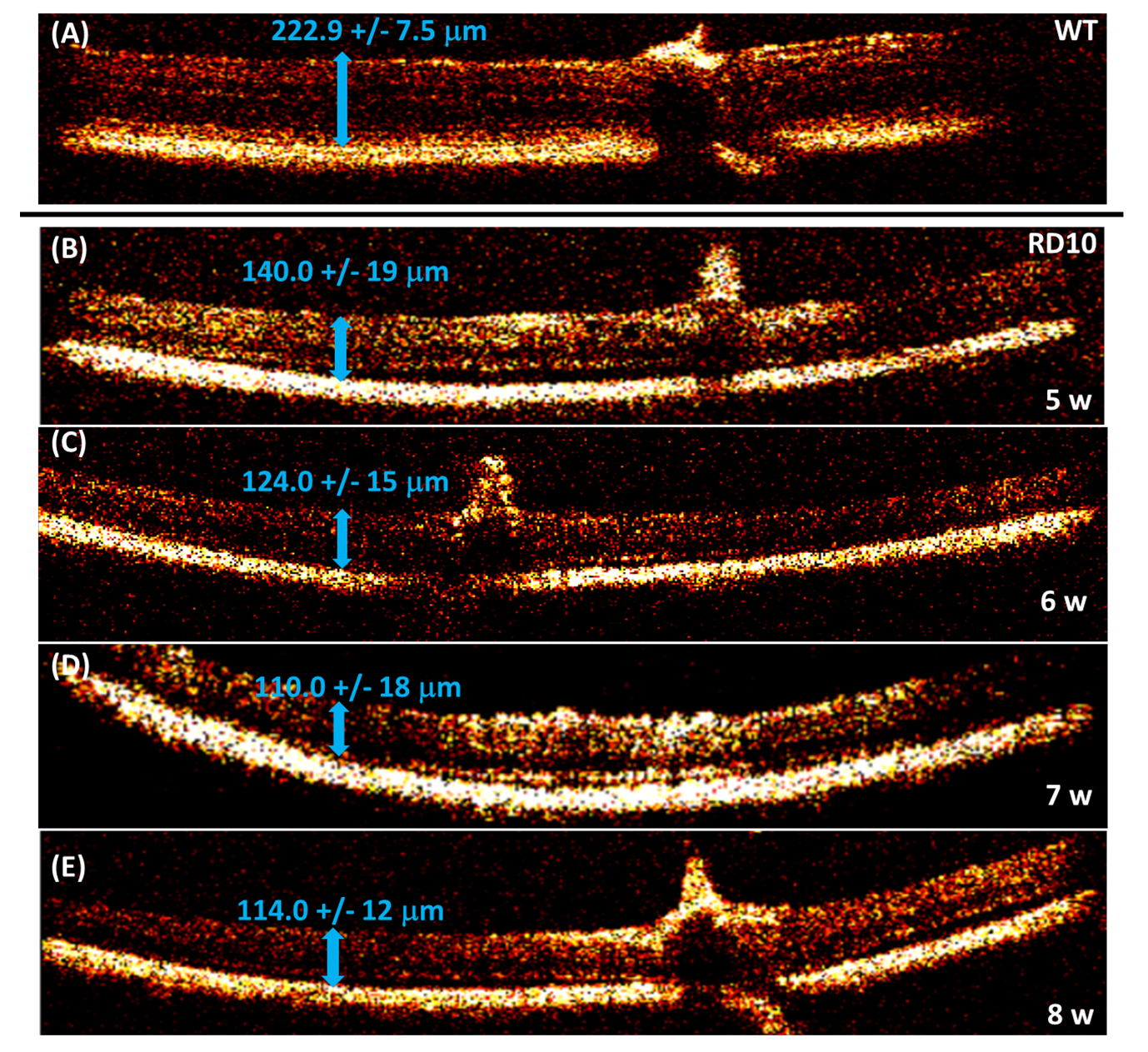


Fig S5. Retinal thickness measured by OCT in Wild type and RD10 mice. There is a clear trend of decrease in retinal thickness in RD10 mice with progression of age.
